# S-Methylcysteine Ameliorates the Intestinal Damage Induced by *Eimeria tenella* Infection *via* Targeting Oxidative Stress and Inflammatory Modulators

**DOI:** 10.3389/fvets.2021.754991

**Published:** 2022-01-05

**Authors:** Ehab Kotb Elmahallawy, Alaa Fehaid, Dina M. M. EL-shewehy, Amany M. Ramez, Abdulsalam A. M. Alkhaldi, Rehab Mady, Nasr Elsayed Nasr, Nagah Arafat, Eman A. A. Hassanen, Khalaf F. Alsharif, Walied Abdo

**Affiliations:** ^1^Department of Zoonoses, Faculty of Veterinary Medicine, Sohag University, Sohag, Egypt; ^2^Forensic Medicine and Toxicology Department, Faculty of Veterinary Medicine, Mansoura University, Mansoura, Egypt; ^3^Zoology Department, Faculty of Science, Mansoura University, Mansoura, Egypt; ^4^Biology Department, College of Science, Jouf University, Sakaka, Saudi Arabia; ^5^Department of Pharmacology, Faculty of Veterinary Medicine, Damanhour University, Damanhour, Egypt; ^6^Biochemistry and Clinical Biochemistry, Faculty of Veterinary Medicine, Kafrelsheikh University, Kafr El-Sheikh, Egypt; ^7^Department of Poultry Diseases, Faculty of Veterinary Medicine, Mansoura University, Mansoura, Egypt; ^8^Department of Parasitology, Faculty of Veterinary Medicine, Zagazig University, Zagazig, Egypt; ^9^Department of Clinical Laboratory Sciences, College of Applied Medical Sciences, Taif University, Taif, Saudi Arabia; ^10^Department of Pathology, Faculty of Veterinary Medicine, Kafrelsheikh University, Kafr El-Sheikh, Egypt

**Keywords:** S-Methylcysteine (SMC), anticoccidial, *Eimeria tenella*, inflammatory, oxidative stress

## Abstract

Avian coccidiosis is one of the major parasitic diseases in the poultry industry. The infection is caused by *Eimeria* species, and its treatment relies mainly on the administration of anticoccidial drugs, which can result in drug resistance and side effects. The recent trends in avian coccidiosis treatment is directed to the development of a new therapy using herbal compounds. S-Methylcysteine (SMC) is considered one of the organosulfur compounds in garlic that showed promising activity in the treatment of different pathological conditions *via* a wide range of anti-inflammatory and antioxidant mechanisms. In this study, the anticoccidial activity of SMC was investigated in *Eimeria tenella*-infected chickens compared to diclazuril as a widely used anticoccidial drug. In this regard, 14-day-old broilers were divided into six groups (*n* = 18). The first group (G1) was the healthy control group, while the second group (G2) was the non-infected SMC group treated at a dose of 50 mg/kg b.w. (high dose). Moreover, the third group (G3) was the positive control group (infected and non-treated). The fourth group (G4) was the infected group treated with SMC of 25 mg/kg b.w. (low dose), while the fifth group (G5) was the infected group treated with SMC of 50 mg/kg b.w. (high dose). Conversely, the sixth group (G6) was the diclazuril-treated group. The anticoccidial effects of SMC and diclazuril were evaluated by counting oocysts and recording the body weight gain, feed conversion ratio, clinical signs, lesions, and mortality rate. Interestingly, SMC showed potent anticoccidial activity, which was exemplified by reduction of oocyst count. Furthermore, the biochemical, antioxidant, and anti-inflammatory parameters in the cecal tissues were restored toward their control levels in G4, G5, and G6. Histopathological observation of cecal tissues was consistent with the aforementioned results revealing the ameliorative effect of SMC against *E. tenella* infection. This study concluded novel findings in relation to the anticoccidial role of SMC as a plant-based compound against the *E. tenella*-induced coccidiosis in broiler chickens combined with its antioxidative and anti-inflammatory properties. Further studies for exploring the mechanistic pathways involved in this activity and the potential benefits from its use in association with conventional anticoccidial drugs are warranted.

## Introduction

The poultry industry is considered an emerging food-producing sector in Egypt, reflecting its role in food security and economic development (1). Coccidiosis is the main reason for low performance and productivity in poultry and is a protozoal infection caused by the genus *Eimeria* (2). *Eimeria* invade the intestinal tract's epithelium, resulting in reduced feed intake (FI) and absorption and secondary bacterial infection (3). Coccidiosis was reported as a major worldwide problem and the top broiler-related disease in the United States in 2019 (4). There are seven species of *Eimeria*: *E. tenella, E. necatrix, E. acervulina, E. maxima, E. brunetti, E. mitis*, and *E. praecox*. They differ in their pathogenicity and site in the intestinal tract (5). Among these, *E. tenella* is considered a strong pathogenic species that induces hemorrhagic cecal coccidiosis in chickens, clinically presenting as bloody diarrhea, severe body weight loss, and death. The life cycle of *E. tenella* is monogenetic and host specific. It undergoes two endogenous phases (schizogony and gametogony) and is initiated by the sporozoite infection of the host cecum, leading to hemorrhagic lesions (6). To control avian coccidiosis, both vaccination and prophylactic anticoccidial drug administration (chemotherapy) are the widely used methods (7, 8). However, the used vaccines, either attenuated or non-attenuated live oocysts, show a risk of reaction development in the vaccinated chickens with different degrees (9). The anticoccidial drugs are used to either kill the coccidial population (coccidiocidal drugs) or prevent coccidial replication and growth (coccidiostatic drugs). To be effective, drugs should be used as a prophylactic, not a therapeutic method (10). Unfortunately, anticoccidial drugs (sulfonamides, pyrimidine derivatives, and polyether ionophores) have shown a problem of drug resistance (11, 12). To prevent drug resistance, alternative treatments should be used, such as aromatherapy, plants extracts, and probiotics. Using natural plant-based extracts as a therapy or supplement is currently targeted to obtain their benefits with minimum side effects compared to chemical therapies. Many plant-based compounds have shown protective effects against coccidiosis by inhibition of *Eimeria* species development, such as the extracts of citric fruits, oregano, mushrooms, and turmeric (13–15). Garlic (*Allium sativum*) is rich in bioactive compounds, which were used as a source of different medicinal drugs (16). One of the organosulfur compounds in garlic is S-methylcysteine (SMC), which was reported in different studies as a treatment for different pathological conditions, including cancer, obesity, and neurological disorders (17–19). SMC exhibited many biological reactions, such as anti-inflammatory and antioxidant effects (20). To date, no previous studies investigated the anticoccidial effect of SMC. Therefore, this study aimed to assess the potential of SMC as a plant-based compound to control avian coccidiosis by recording the body weight gain (BWG), feed conversion ratio (FCR), clinical signs, lesions, and mortality rate following the infection and treatment compare with control groups. Furthermore, oocysts were counted; the biochemical, antioxidant, and anti-inflammatory parameters were measured; and the histopathological changes in cecal tissues in response to treatment by SMC were recorded.

## Materials and Methods

### Ethical Approval

This study followed the guidance of the Research, Publication, and Ethics Committee of the Faculty of Veterinary Medicine, Kafrelsheikh University, Egypt (ethical approval number: KFS-2019/3).

### Chemicals

All chemicals and reagents used, including SMC, were purchased from Sigma-Aldrich, Egypt. Diclazuril (Diclosol^®^ suspension 10 mg/ml) was purchased from Pharma Swede—Egypt and administered in drinking water at a concentration of 2.5 ppm (1 ml of Diclosol^®^/4 L of drinking water).

### Birds

Chicks (1 day old, unsexed) of Cobb strain broilers were obtained from a hatchery in Kafr El Sheikh, Egypt. Rearing was initiated in floor pens till 7 days old; then, chickens were moved to wire-floored cages until the end of the experiment following all hygienic measures. Balanced commercial ration free from antibiotics and anticoccidials and tap water were provided to chickens *ad libitum*. Optimum temperature was adjusted using electric radiators and ventilators. Regular examinations of fecal samples were conducted twice daily during the first 14 days of the experiment (before the experimental infection) to confirm the privation of *Eimeria* oocysts by flotation technique using NaCl saturated solution with specific gravity of 1.28. Chickens were vaccinated against Newcastle disease by eye drops of Hitchner B1 strain in drinking water at 7 days old. Moreover, chickens were immunized against infectious bursal disease by Gumboro vaccine at 12 days old.

### *Eimeria tenella* Oocysts

Oocysts of *E. tenella* were isolated from cecal tissues of naturally infected birds following the Chapman and Shirley method (21). Isolated oocysts were then maintained in 4-week-old coccidia-free Cobb strain broilers through two passages at 3-month intervals. The doses used per bird were 4,000 oocysts/1 ml phosphate-buffered saline (PBS) for the first passage and 8,000 oocysts/1 ml PBS for the second passage. Subsequently, oocysts were collected and kept in solution of 2.5% potassium dichromate at 26–28°C for sporulation. The McMaster technique was used to clear and count the sporulated oocysts (22), which were then kept in 2.5% potassium dichromate at 4°C for a maximum of 4 weeks to be used in the experimental infection.

### Experimental Protocol

At the age of 14 days, 108 chicks were divided randomly into six groups (*n* = 18). Then, each group was subdivided into three different cages (*n* = 6); each cage represents a biological replication. The six main groups were as follows:

Group 1 (G1): negative control group; non-infected and non-treated chicks.

Group 2 (G2): SMC-treated group; non-infected chicks and treated at a dose of 50 mg/kg b.w., in drinking water.

Group 3 (G3): positive control group; infected and non-treated chicks.

Group 4 (G4): low-dose SMC-treated group; infected chicks and treated at a dose of 25 mg/kg b.w., in drinking water.

Group 5 (G5): high-dose SMC-treated group; infected chicks and treated at a dose of 50 mg/kg b.w. SMC, in drinking water.

Group 6 (G6): diclazuril-treated group; infected chicks and treated with 2.5 ppm diclazuril in drinking water.

At the age of 14 days, all groups, except G1 and G2, were infected with 4.0 × 10^4^ sporulated oocysts/chick of *E. tenella* in 1 ml of PBS. Disposable Pasteur pipettes were used for the inoculation directly into the crop. On the 2nd day after infection (at the age of 15 days), SMC and diclazuril were administered for 4 consecutive days.

### Sampling

**Figure 1:**
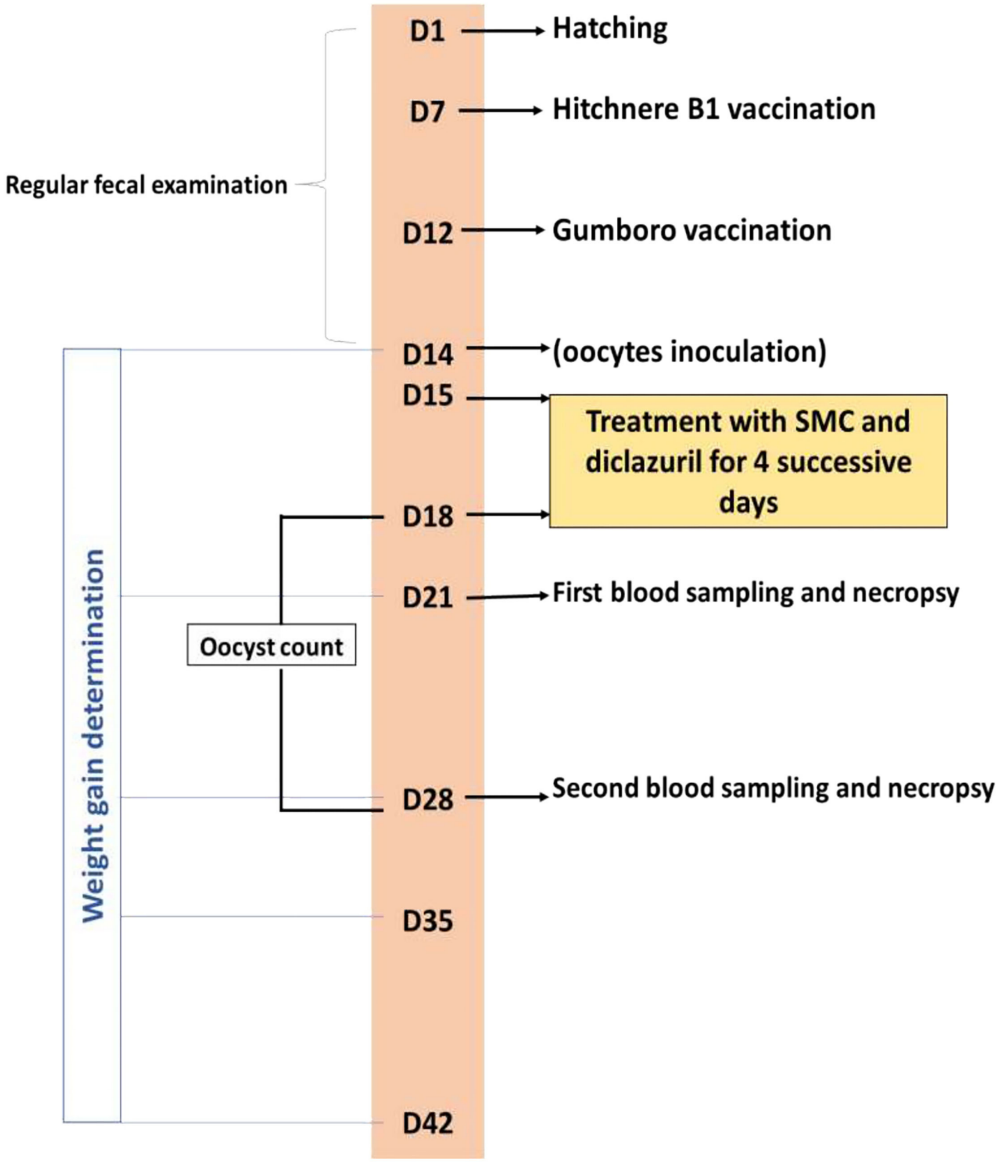
Timeline of the experimental protocol explaining the day (D) number, treatments, vaccination, and sampling.

Blood samples were drawn from the wing vein of five chicks of each group on the 7th and 14th day after infection. Blood was used to separate the serum samples, which were kept at −20°C for further biochemical analysis. The same five chicks were killed by neck dislocation, and both the liver and cecum were collected and trimmed. Both organs were divided into two portions for the antioxidant's assessment and histopathological examination. The experimental treatments and sampling timeline are presented in Figure 1.

### Evaluation of Anticoccidial Efficacy of SMC

Clinical signs of coccidia infection, including bloody diarrhea, anorexia, and depression, were observed and recorded after infection in the SMC-treated groups compared to the untreated groups. Moreover, the growth performance was evaluated by recording the chicken's body weights at 14, 21, 28, 35, and 42 days of age. FI, BWG, and FCR were recorded as described previously (23). Moreover, *E. tenella* oocysts were counted using the modified McMaster technique (22) starting from the 4th day after infection until the 16th day. Mortality percentages and gross lesions were scored on the 7th and 14th day according to Johnson and Reid (24).

### Histopathological Examination

On the 7th and 14th day after infection, cecal tissue samples were obtained from the slaughtered chickens. Collected samples were rinsed with neutral PBS and fixed in 10% formalin. Subsequently, tiny blocks were trimmed and processed in an automatic processor containing many chambers with ascending concentrations of alcohol to dehydrate the tissue before paraffinization. Then, 3- to 5-micron-thick blocks were cut using a microtome, stained with hematoxylin and eosin, and allowed to dry before the light microscopical analysis.

### Biochemical Parameters Analysis

In serum samples, aspartate, and alanine amino transferases (AST and ALT, respectively) were measured following Varliy's protocol (25). Moreover, alkaline phosphatase (ALP), total protein (TP), and albumin contents were measured according to the methods of Thirunavukkarasu et al. (26), Domas (27), and Doumas et al. (28), respectively. Serum creatinine and uric acid levels were measured according to the methods of Henry (29) and Haisman and Muller (30), respectively.

### Antioxidative Parameter Analysis

Liver tissues were dissected and cleaned using neutral pH 50 mM Tris–HCl and then homogenized in an ice-cold solution containing neutral 50 mM Tris–HCl. Homogenates were used for the antioxidative parameter analysis after centrifugation at 2,500 rpm for 30 min. The enzymatic activities of hepatic superoxide dismutase (SOD), glutathione peroxidase (GSHPx), and catalase (CAT) were measured in liver homogenates depending on the methods of Nishikimi et al. (31), Paglia (32), and Aebi (33), respectively.

### Gene Expression Analysis of Inflammatory Parameters

Reverse transcription-quantitative polymerase chain reaction (RT-qPCR) was used to analyze the gene expressions. Total RNA from the liver was extracted using the TRIzol^®^ reagent (Invitrogen, USA). Then, reverse transcription of the extracted RNA was conducted to create cDNA. Table 1 shows the employed primer sequences of the housekeeping (β-actin), interleukin 1 β (IL-1β), interferon gamma (IFN-γ), and nuclear factor kappa-light-chain-enhancer of activated B cell 1 (NF-κB1) genes. RT-qPCR reactions were completed by Power SYBR^®^ Green PCR Master Mix (Applied Biosystems, USA). Reactions were monitored by the 7500 Real-Time PCR System (Applied Biosystems, USA). Thermal cycles were performed at 95°C for 4 min, 40 cycles of 10 s at 95°C, 30 s at 60°C, and finally 10 s at 72°C. Data were presented as relative fold changes compared to control's gene expressions.

**Table 1:** Primers used for RT-qPCR analysis.

**Gene**	**Primer**	**References**
β-actin	F: ACCTGAGCGCAAGTACTCTGTCT R: CATCGTACTCCTGCTTGCTGAT	NM_205518.1 (34)
NF-κB1	F: TACCGGGAACAACACCACTG R: CAGAGGGCCTTGTGACAGTA	NM_205134 (35)
IFN-γ	F: GAACTGGACAGAGAGAAATGAGA R: ATGTGTTTGATGTGCGGCTT	NM_205149 (35)
IL-1β	F: CAGCCTCAGCGAAGAGACCTT R: CACTGTGGTGTGCTCAGAATCC	XM_015297469.1 (36)

### Statistical Analysis

GraphPad Prism 5 software was used to analyze the data by one-way analysis of variance and Tukey multiple comparisons. Data were presented as means ± SD. Statistical difference was considered significant when *p*-value is < 0.05.

## Results

### Direct Anticoccidial Effect of SMC

#### Clinical Signs, Lesion Scoring, and Mortality Rate

As presented in Table 2, data showed that there were no observed abnormalities or recorded lesions and deaths in both control chickens (G1) and SMC-treated group (G2). In contrast, the infected non-treated group (G3) had typical clinical signs of coccidiosis showing dullness, reduced appetite, depression, wasting, bloody diarrhea, and increased weakness with progression resulting in death within 4–5 days of infection, whereas five of 18 chickens died in G3. After treatment of the infected chicken with high dose of SMC (G5), only one bird died, while the low-dose SMC treatment resulted in death of three birds. Diclazuril treatment resulted in death of two chickens. The scored lesions were significantly reduced in chickens treated with high-dose SMC and diclazuril (G5 and G6) with a higher significance than the reduction induced by the low dose of SMC (G4) after 7 days of infection, as shown in Table 2. Moreover, after 14 days of infection, treatment with high dose of SMC showed the highest significant improvement in the lesion's score. These data suggested the protective effect of SMC against coccidiosis symptoms and mortality rate.

**Table 2:** The effect of SMC on mortality % and lesion score in broiler chickens experimentally infected with *E. tenella*.

**Parameter**	**Control**	**SMC**	**Infected**			
			**Non-treated**	**SMC low dose**	**SMC high dose**	**Diclazuril**
Number of birds	18	18	18	18	18	18
Mortality	0	0	5	3	1	2
Percent	0	0	27.78	16.67	5.55	11.11
Lesion's score (7 days)	0	0	3.70 ± 0.29	2.40 ± 0.48^a^	1.20 ± 0.29^ab^	1.70 ± 0.29^ab^
Lesion's score (14 days)	0	0	2.70 ± 0.29	1.40 ± 0.29^a^	0.40 ± 0.41^ab^	1.00 ± 0.70^a^

*Data are expressed as mean ± S.D. a and b indicate statistical significance in comparison with the infected non-treated group (G3) and SMC treated at low dose (G4). Means within rows with different superscripts differ at p ≤ 0.05*.

### Assessment of Growth Performance

To assess the growth performance, BWGs of all groups were measured and presented in Table 3. All treated groups (G4, G5, and G6) showed significant improvements in the BWG compared to the non-treated group (G3). The highest improvements were induced by the high dose of SMC (G5), which showed no significant changes compared to the control healthy chickens (G1) at 21 days of age. In addition to the BWG, FCRs were measured and presented in Table 4. Chickens treated with high dose of SMC showed the best significant improvements in the FCRs compared to the other treated and the non-treated groups. Interestingly, the SMC-exposed group (G2) had no significant changes in the BWG and FCRs compared to the healthy control group (G1), indicating that SMC has no side effects. The assessment of growth performance suggested that SMC had a protective role against the coccidiosis-induced reduction in the growth rate.

**Table 3:** The effect of SMC on body weight gain (in grams) in broiler chickens experimentally infected with *E. tenella*.

**Days of age**	**Control**	**SMC**	**Infected**			
			**Non-treated**	**SMC low dose**	**SMC high dose**	**Diclazuril**
14th−21st	349.23 ± 7.86	357.78 ± 12.25	138.34 ± 11.58^a^	236.37 ± 12.14^ab^	307.21 ± 6.87^abcd^	270.08 ± 11.75^abc^
21st−28th	376.64 ± 9.42	418.43 ± 5.62	211.29 ± 10.96^acd^	295.70 ± 12.43^abcd^	360.20 ± 26.33^bcd^	317.39 ± 10.34^ab^
28th−35th	357.13 ± 26.77	368.35 ± 17.25	271.51 ± 20.84^ab^	305.40 ± 13.28^ab^	337.30 ± 11.57^bc^	311.55 ± 7.45^ab^
35th−42nd	406.90 ± 21.09	447.00 ± 12.99	304.00 ± 5.88^a^	323.50 ± 14.90^ab^	408.00 ± 6.43^bc^	367.00 ± 25.19^ab^

*Data are expressed as mean ± S.D. a, b, c, and d indicate statistical significance in comparison with control, infected non-treated, low dose SMC-treated, and diclazuril-treated groups, receptively. Means within rows with different superscripts differ at p ≤ 0.05*.

**Table 4:** The effect of SMC on feed conversion ratio (in grams) in broiler chickens experimentally infected with *E. tenella*.

**Days of age**	**Control**	**SMC**	**Infected**			
			**Non-treated**	**SMC low dose**	**SMC high dose**	**Diclazuril**
14th−21st	2.28 ± 0.10	2.16 ± 0.14	4.84 ± 0.17^a^	3.26 ± 0.24^ab^	2.75 ± 0.17^abcd^	3.22 ± 0.11^ab^
21st−28th	2.48 ± 0.11	2.25 ± 0.18	4.25 ± 0.18^a^	2.91 ± 0.11^ab^	2.43 ± 0.26^bcd^	3.04 ± 0.17^ab^
28th−35th	2.57 ± 0.08	2.31 ± 0.16	3.75 ± 0.19^a^	2.69 ± 0.26^b^	2.36 ± 0.13^b^	2.54 ± 0.27^b^
35th−42nd	2.14 ± 2.25	2.30 ± 2.09	2.78 ± 2.8^a^	2.16 ± 2.44	2.10 ± 2.36^b^	2.60 ± 2.51

*Data are expressed as mean ± S.D. a, b, c, and d indicate statistical significance in comparison with control, infected non-treated, low dose SMC-treated, and diclazuril-treated groups, receptively. Means within rows with different superscripts differ at p ≤ 0.05*.

### Count of Oocysts

On the 4th day after infection, *E. tenella* oocysts started to appear in G3, and the highest count was recorded on the 8th day. Then, the count decreased gradually until the 14th day after the infection, as shown in Table 5. In all infected and treated groups (G4, G5, and G6), the oocyst count reduced significantly compared to the infected non-treated G3. Interestingly, treatment with high-dose SMC (G5) induced the highest significance compared to other treatments (G4 and G6). The recorded oocyst count reveals the protective effect of SMC against coccidiosis by reducing the oocyst count.

**Table 5:** The effect of SMC on an oocyst count (×10^6^/g of feces) from the 4th to 14th day post infection in broiler chickens experimentally infected with *E. tenella* oocysts.

**Days post-infection**	**Control (×10^6^/g of feces)**	**SMC (×10^6^/g of feces)**	**Infected**			
			**Non-treated (×10^6^/g of feces)**	**SMC low dose (×10^6^/g of feces)**	**SMC high dose (×10^6^/g of feces)**	**Diclazuril (×10^6^/g of feces)**
4th day	0.00 ± 0.00	0.00 ± 0.00	0.23 ± 34.38	0.12 ± 20.33^a^	0.10 ± 14.91^a^	0.16 ± 21.58^a^
5th day	0.00 ± 0.00	0.00 ± 0.00	0.75 ± 46.04	0.40 ± 29.59^a^	0.29 ± 28.81^ab^	0.34 ± 30.09^ab^
6th day	0.00 ± 0.00	0.00 ± 0.00	1.38 ± 19.75	0.77 ± 132.59^a^	0.47 ± 53.74^abc^	0.67 ± 43.31^a^
7th day	0.00 ± 0.00	0.00 ± 0.00	1.78 ± 94.45	0.99 ± 88.78^a^	0.62 ± 38.50^abc^	0.87 ± 49.44^ab^
8th day	0.00 ± 0.00	0.00 ± 0.00	1.50 ± 80.04	0.82 ± 60.76^a^	0.56 ± 64.52^abc^	0.88 ± 57.44^a^
9th day	0.00 ± 0.00	0.00 ± 0.00	0.99 ± 105.82	0.67 ± 38.54^a^	0.47 ± 45.27^ab^	0.56 ± 85.22^a^
10th day	0.00 ± 0.00	0.00 ± 0.00	0.64 ± 58.00	0.47 ± 38.44^a^	0.26 ± 25.54^abc^	0.40 ± 58.11^a^
11th day	0.00 ± 0.00	0.00 ± 0.00	0.58 ± 30.10	0.37 ± 20.09^a^	0.21 ± 39.81^abc^	0.31 ± 36.90^ab^
12th day	0.00 ± 0.00	0.00 ± 0.00	0.46 ± 38.63	0.23 ± 16.04^a^	0.18 ± 24.01^abc^	0.24 ± 30.07^a^
13th day	0.00 ± 0.00	0.00 ± 0.00	0.40 ± 10.64	0.31 ± 26.52^a^	0.14 ± 17.82^abc^	0.22 ± 21.83^a^
14th day	0.00 ± 0.00	0.00 ± 0.00	0.25 ± 34.90	0.16 ± 21.25^a^	0.10 ± 7.95^abc^	0.13 ± 6.52^a^

*Oocyst counts are shown as (no. ×10^6^/g of feces) and the data are expressed as mean ± S.D. a, b, and c indicate statistical significance in comparison with infected non-treated, low dose SMC-treated, and diclazuril-treated groups, receptively. Means within rows with different superscripts differ at p ≤ 0.05*.

### Histopathology

**Figure 2:**
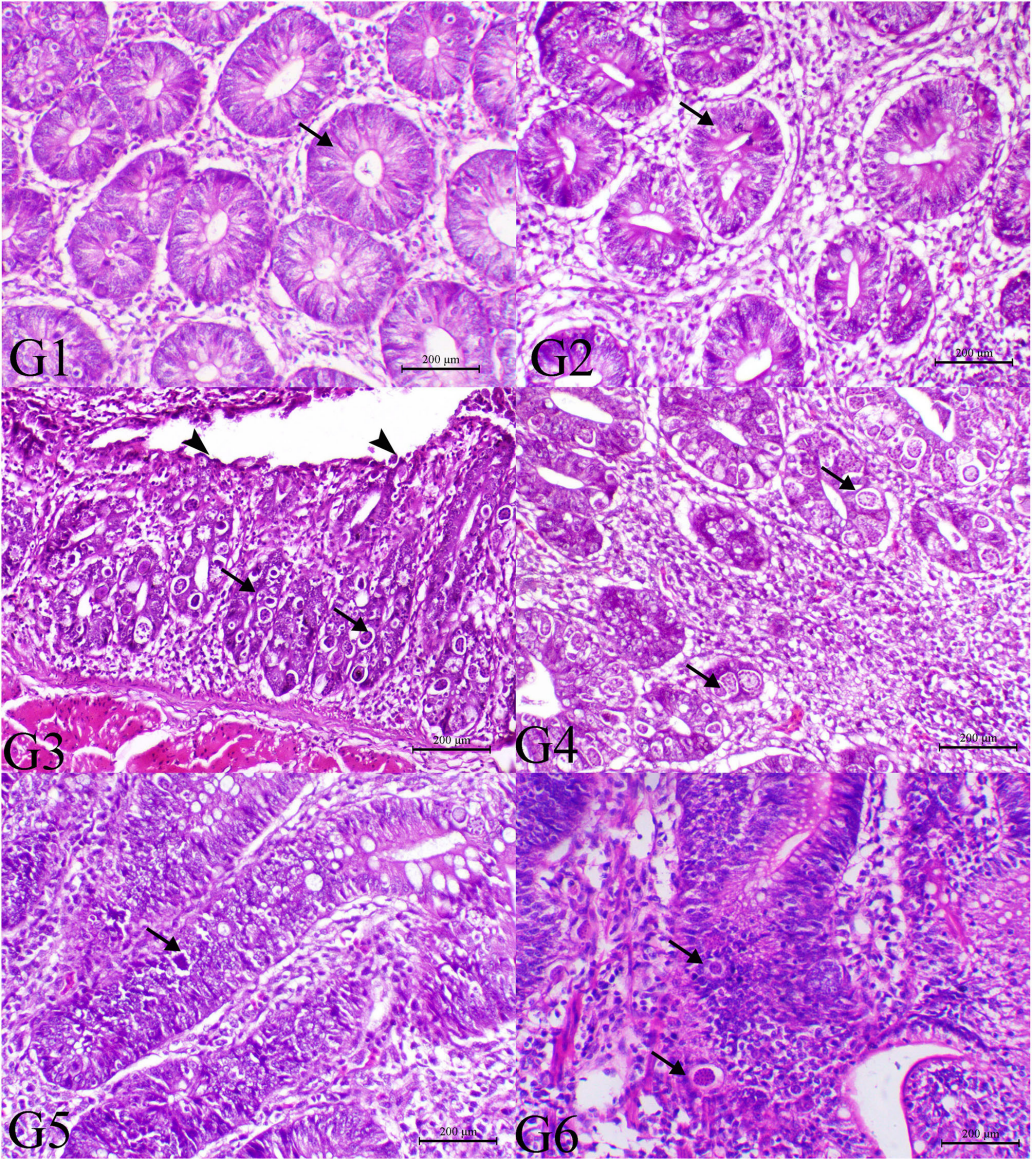
Histopathology of cecal tissues of the normal bird (G1), SMC-treated birds (G2), *E. tenella*-challenged birds and examined on the 7th day post infection (G3), challenged and treated birds with SMC low dose (G4), high dose (G5), and diclazuril (G6). G1 and G2 (arrows indicate normal intestinal crypts), G3 (arrowheads indicate desquamation of the intestinal epithelium and arrows reveal severe infection of the intestinal crypts with parasitic schizonts), G4 (arrow indicates decrease the parasites within the intestinal mucosa), G5 (arrow indicates very few parasites), and G6 (arrow indicates moderate number of coccidial parasitic stages), H&E stain, bar = 200 μm.

**Figure 3:**
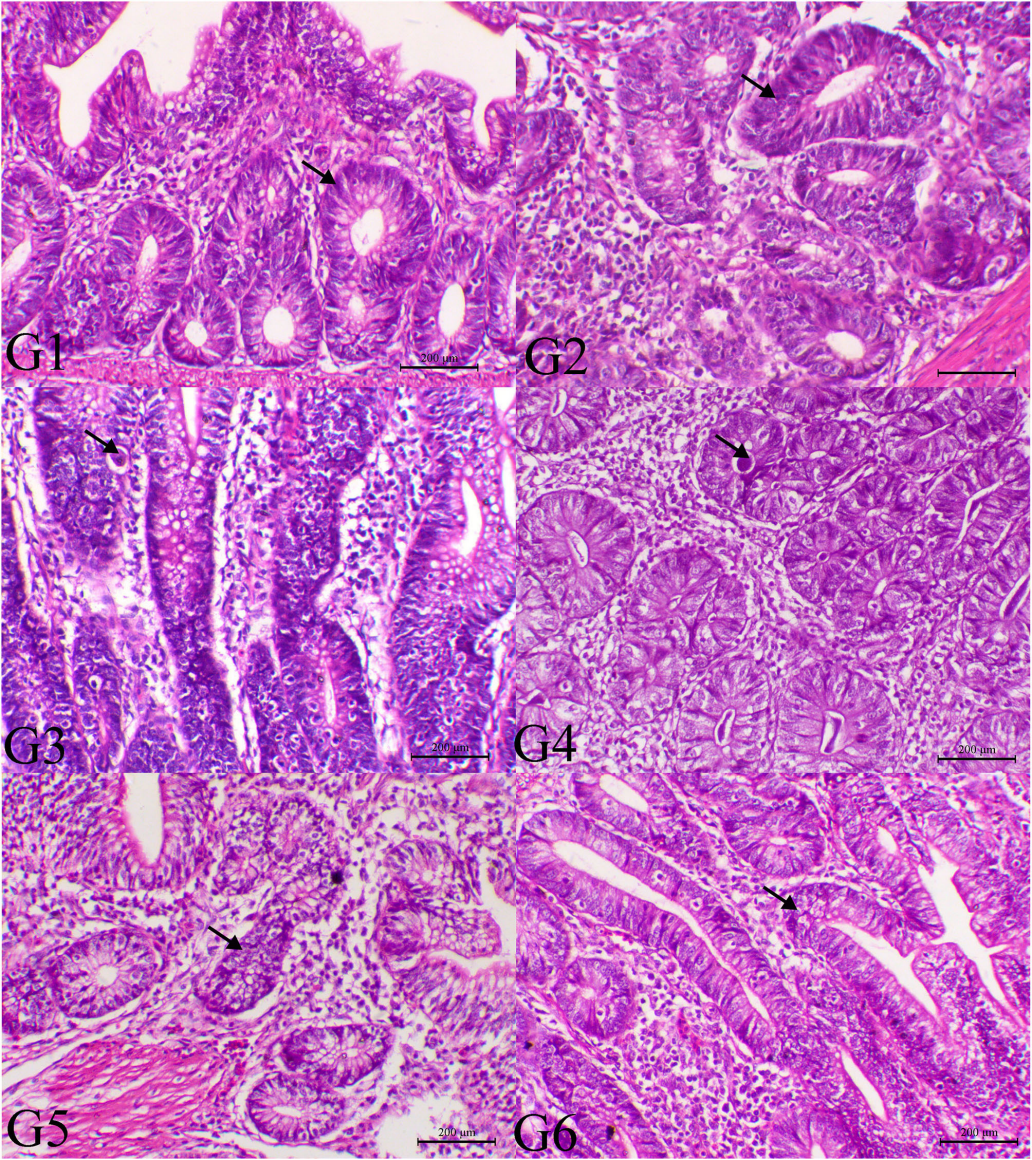
Histopathology of cecal tissues of the normal bird (G1), SMC-treated birds (G2), *E. tenella*-challenged birds and examined on the 14th day post infection (G3), challenged and treated birds with SMC low dose (G4), high dose (G5), and diclazuril (G6). G1 and G2 (arrows indicate normal intestinal crypts), G3 (arrow reveals the presence of parasites within the intestinal crypts), G4 (arrow indicates marked decrease of the parasites within the intestinal mucosa), G5 (arrow indicates normal intestinal crypts), and G6 (arrow indicates hyperplastic changes within the intestinal mucosa), H&E stain, bar = 200 μm.

The histopathological findings of posterior intestinal segments are shown in Figure 2. The cecal segments of birds of G1 and G2 showed normal intestinal mucosal lining with normal glandular epithelium. However, the ceca of the G3 revealed severe necrotic enteritis associated with marked infestation of the intestinal glands with schizont of the coccidial parasites. The diseased birds treated with low dose of SMC showed a decrease in necrotic changes within the glandular epithelium. The high dose showed a marked decrease in degenerative changes associated with a marked decrease in infestation of glandular epithelium with coccidial stages. Diseased birds treated with diclazuril revealed a decrease in necrotic change with a decrease in parasitic stages. The pathological findings of the second sacrifice are presented in Figure 3. During the 2nd sacrifice of the study, the diseased birds showed a decrease in the number of parasites within the intestinal epithelium with an obvious chronic inflammatory reaction and hyperplastic changes of the intestinal epithelium. Diseased birds treated with diclazuril revealed a marked decrease in coccidial stages and intestinal inflammatory changes. Interestingly, the high dose of SMC revealed marked decrease of the enteritis and obvious increase of the regenerative and reparative actions within the mucosal epithelium with an increase in intraepithelial lymphocytes.

### Indirect Protective Effect of SMC

#### Assessment of Biochemical Parameters in the Serum

As presented in Tables 6, 7, biochemical parameters were measured after 7 and 14 days of the infection. The results revealed that G3 showed significant increases in ALT, AST, ALP, creatinine, and uric acid levels and significant decreases in TP and albumin levels compared to G1. In G4, G5, and G6, treatment with SMC (low and high doses) and diclazuril could significantly improve the levels of the biochemical parameters compared to those in G3. These data suggested the protective effect of SMC on the disturbed biochemical parameters by the effect of *Eimeria* infection.

**Table 6:** The effect of SMC on the serum biochemical parameters (values are means ± SD) in broiler chickens experimentally infected with *E. tenella* at 7th day post infection.

**Parameter**	**Non-infected**		**Infected**			
	**Control G1**	**SMC G2**	**Non-treated G3**	**SMC low dose G4**	**SMC high dose G5**	**Diclazuril G6**
ALT (U/L)	10.33 ± 1.24^e^	13.33 ± 1.24^a^	19.33 ± 5.24^b^	20.33 ± 3.68^c^	19 ± 0.81^d^	19 ± 2.16^d^
AST (U/L)	158.67 ± 25.3^e^	170.33 ± 15.45^a^	278.33 ± 31.56^b^	236 ± 36.24^c^	198.33 ± 8.37^d^	200 ± 8.16^d^
ALP (U/L)	*1, 908*±274.1^e^	*1, 907*±210.77^a^	4266.7 ± 839.73^b^	*2, 350*±515.2^c^	2053.66 ± 585.71^d^	1944.33 ± 189.58^d^
T P (g/dl)	5.26 ± 0.66^a^	4.53 ± 0.41^e^	3.23 ± 0.26^d^	3.66 ± 0.20^c^	4.06 ± 0.09^b^	4.16 ± 0.17^b^
Albumin (g/dl)	1.63 ± 0.13^a^	1.72 ± 0.22^e^	1.21 ± 0.08^d^	1.33 ± 0.09^c^	1.53 ± 0.12^b^	1.73 ± 0.17^b^
Creatinine (mg/dl)	0.50 ± 0.08^e^	0.46 ± 0.04^a^	0.85 ± 0.04^b^	0.60 ± 0.08^c^	0.50 ± 0.08^cde^	0.60 ± 0.08^cde^
Uric acid (mg/dl)	6.78 ± 0.71^e^	6.78 ± 0.76^a^	9.76 ± 0.69^b^	7.64 ± 3.09^c^	6.69 ± 0.85^de^	7.63 ± 0.47^de^

*Means within rows with different superscripts differ at p ≤ 0.05. a, b, c, and d indicate statistical significance in comparison with control, infected non-treated, low dose SMC-treated, and diclazuril-treated groups, receptively. Means within rows with different superscripts differ at p ≤ 0.05*.

**Table 7:** The effect of SMC on the serum biochemical parameters (values are means ± SD) in broiler chickens experimentally infected with *E. tenella* at 14th day post infection.

**Parameter**	**Non-infected**		**Infected**			
	**Control G1**	**SMC G2**	**Non-treated G3**	**SMC low dose G4**	**SMC high dose G5**	**Diclazuril G6**
ALT (U/L)	6.00 ± 1.41^e^	5.66 ± 0.47^a^	14.66 ± 1.24^b^	5.00 ± 0.81^c^	7.00 ± 0.81^d^	5.33 ± 0.94^d^
AST (U/L)	213.67 ± 7.76^e^	193.66 ± 7.36^a^	284.33 ± 05.31^b^	223.66 ± 3.39^c^	229.33 ± 18.08^d^	192 ± 12.83^d^
ALP (U/L)	1623.3 ± 107.28^e^	1623 ± 128.83^a^	2,277 ± 189.64^b^	1,491 ± 91.17^c^	1.353 ± 88.58^d^	1424.33 ± 68.66^d^
T P (g/dl)	5.00 ± 0.81^a^	5.33 ± 0.47^e^	4.06 ± 0.16^d^	5.00 ± 0.81^c^	5.53 ± 0.41^b^	4.73 ± 0.52^b^
Albumin (g/dl)	1.53 ± 0.17^a^	1.83 ± 0.09^e^	1.76 ± 0.20^d^	1.93 ± 0.18^c^	1.96 ± 0.19^b^	1.76 ± 0.12^b^
Creatinine (mg/dl)	0.56 ± 0.04^e^	0.53 ± 0.04^a^	0.70 ± 0.08^b^	0.56 ± 0.04^c^	0.56 ± 0.04^cde^	0.55 ± 0.05^cde^
Uric acid (mg/dl)	7.08 ± 1.93^e^	6.08 ± 0.33^a^	6.08 ± 0.33^a^	6.18 ± 0.82^c^	8.07 ± 1.14^de^	8.07 ± 1.14^de^

*Means within rows with different superscripts differ at p ≤ 0.05. a, b, c, and d indicate statistical significance in comparison with control, infected non-treated, low dose SMC-treated, and diclazuril-treated groups, receptively. Means within rows with different superscripts differ at p ≤ 0.05*.

#### Assessment of Antioxidant Parameters

**Figure 4:**
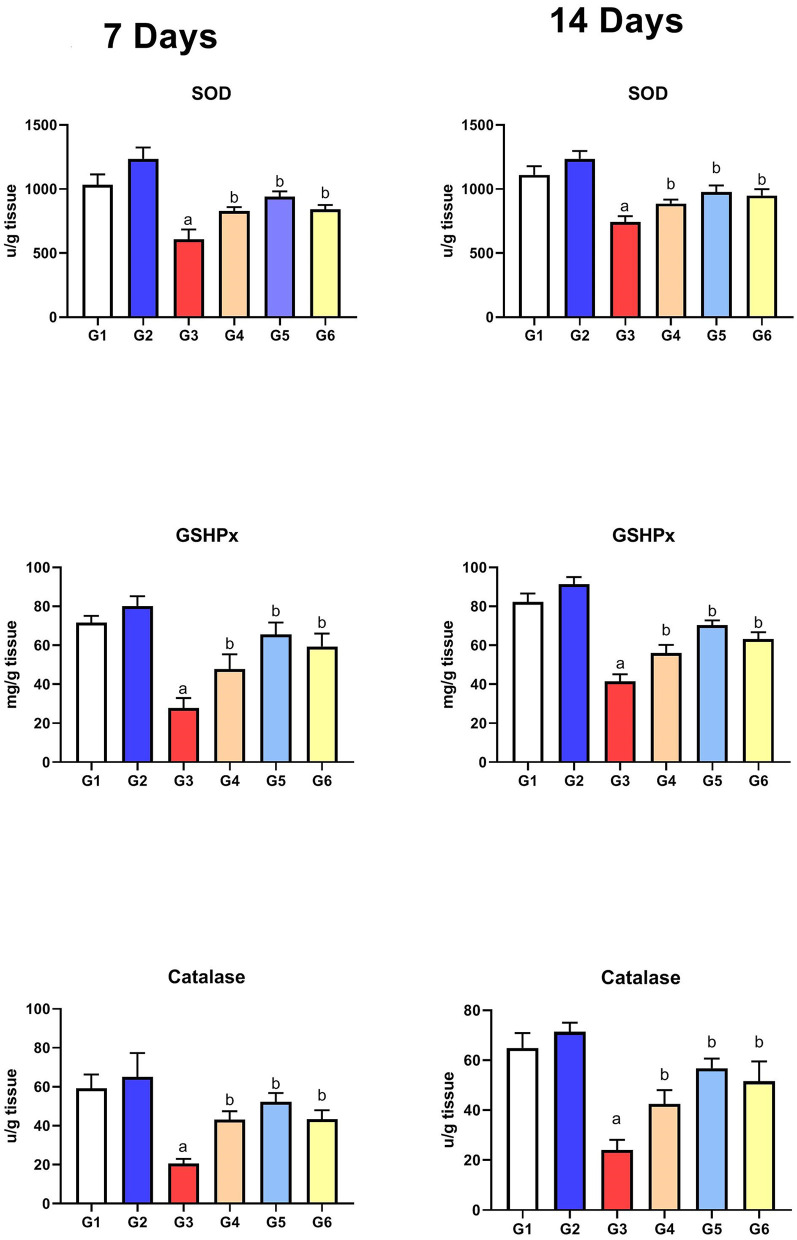
The effect of SMC on the antioxidant parameters on the 7th and 14th day post infection in broiler chickens experimentally infected with *E. tenella*. a and b indicate statistical significance (*p* ≤ 0.05) in comparison with control (G1) and infected non-treated (G3) groups, receptively.

As presented in Figure 4, the levels of the antioxidant enzymes (SOD, GSHPx, and CAT) were significantly reduced in G3 compared to those in G1 on the 7th and 14th day of infection. After treatment with SMC and diclazuril, the enzyme levels showed significant improvement (G4, G5, and G6) compared to the non-treated chickens (G3). Moreover, G2 did not show any significant changes compared to the control group (G1), revealing the antioxidant role of SMC.

#### Assessment of Inflammatory Parameters

**Figure 5:**
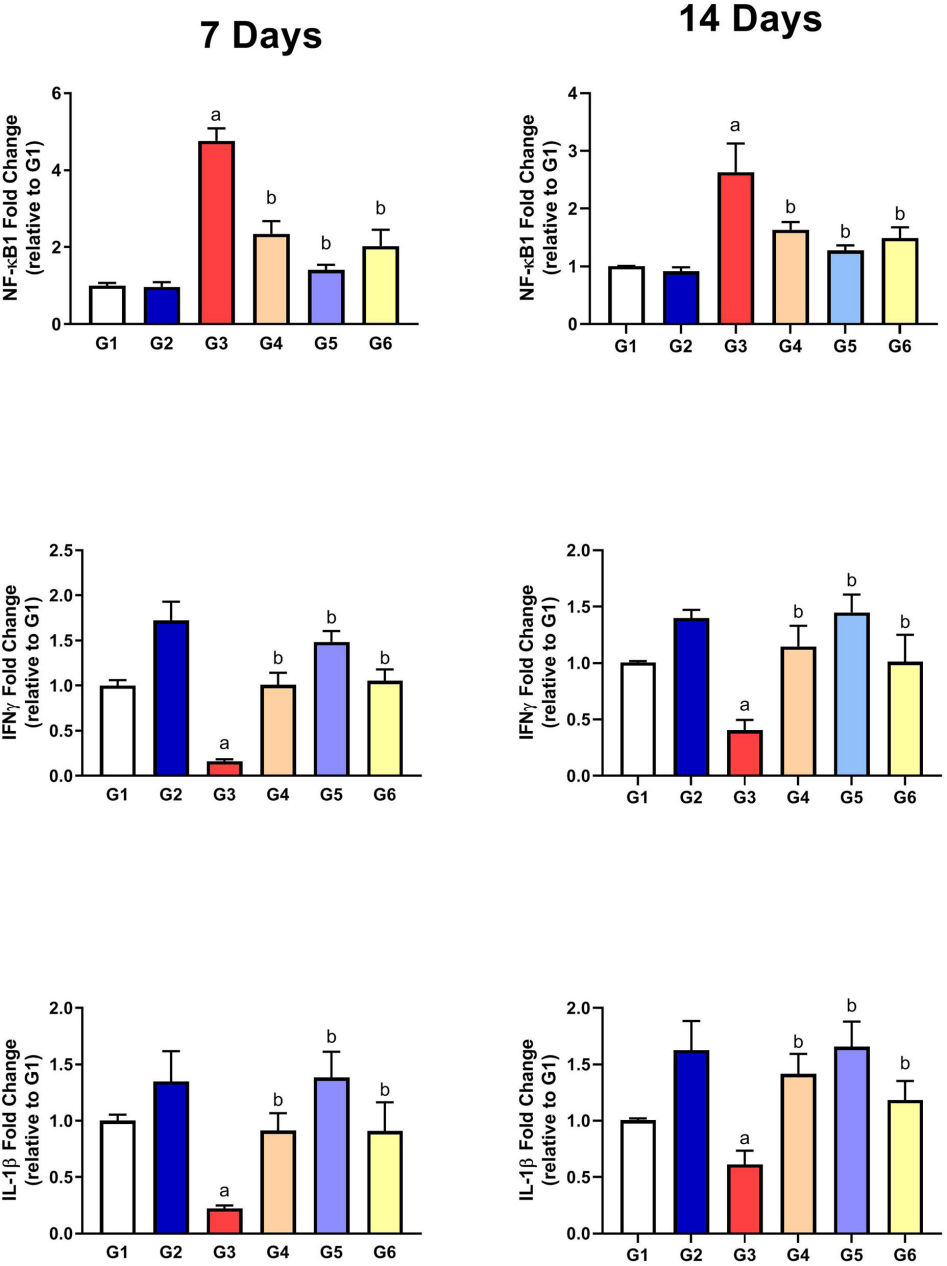
The effect of SMC on the inflammatory parameters on the 7th and 14th day post infection in broiler chickens experimentally infected with *E. tenella*. a and b indicate statistical significance (*p* ≤ 0.05) in comparison with control (G1) and infected non-treated (G3) groups, respectively.

As presented in Figure 5, the gene expressions of IL-1β and IFN-γ were significantly downregulated along with upregulation of NF-κB1 gene expression in G3 compared to G1 on the 7th and 14th day of infection. After treatment with SMC and diclazuril, the dysregulated gene expressions showed significant improvements in G4, G5, and G6 compared to those in G3. These data showed the anti-inflammatory effect of SMC, which is the same effect of diclazuril on the inflammatory response.

## Discussion

Poultry coccidiosis has a great economic significance because of its effect on the production, which is indicated by the low growth rate, high mortalities, and costs of treatment and control (37). Anticoccidial drugs could be classified into four categories depending on their mode of action (38): The first category is drugs that affect an essential cofactor disturbing the biochemical pathway of the coccidia cells, such as sulfonamides and pyrimethamine (39). The second category is drugs that affect the mitochondrial function and inhibit the respiration of coccidian, such as quinolone, meticlorpindol, nicarbazin, and triazinetrione drugs (40). The third category consists of drugs that affect the cell membrane, inducing an osmotic damage, such as ionophores (41). The last category has unknown mechanism of action, such as diclazuril and halofuginone, which inhibit different stages of coccidia differentiation (42). Despite the availability of many anticoccidial drugs, the wide range of their use had developed a loss of sensitivity and high resistance, either quickly, such as quinolones, or after long-term use, such as ionophores (43). Therefore, using plant-based compounds to control the poultry coccidiosis would help avoid the chemical therapy's resistance. Garlic extract compounds, such as SMC, were reported previously to have an antiprotozoal effect against the *Cryptosporidium parvum* infection in mice (44). In this study, the anticoccidial effect of SMC was investigated in poultry for the first time and compared to diclazuril's anticoccidial activity.

In this study, broiler chickens were infected with *E. tenella*, and the infection was confirmed by the oocyst count from the 4th day until the 14th day after infection. SMC affected the oocyst count in a dose-dependent manner, whereas the high dose could reduce the count to a greater extent than the low dose. Moreover, the high dose of SMC had a significant protective effect compared to the diclazuril as a widely used anticoccidial drug. Reduction in the oocyst count by SMC as an organosulfur compound is mostly due to the ability of thiosulfinates to inhibit the microorganism's thiol-containing enzymes, resulting in its antimicrobial effect (45). Furthermore, the allicin content of garlic can inhibit the parasitic RNA synthesis and activate the natural killer immune cells (46). The anticoccidial effect of diclazuril is noted in its ability to disturb the different stages of the *Eimeria* species life cycles by inhibiting the amylopectin synthesis, which is a main component of the cell wall of *E. tenella* parasitic stage (47). Furthermore, diclazuril downregulates the microneme genes, leading to reduction in the second-generation merozoites of *E. tenella* (48), and the enzyme expressions that are responsible for the *E. tenella* cell replication (6). Epithelial cells with *E. tenella* first-generation schizonts are present in the cecal lamina propria and release themselves to develop the second-generation schizonts, which then migrate to the mucosa for the intracellular maturation (49). Here, SMC-treated chickens showed necrotic *E. tenella* schizonts in cecal crypts with migrating cell-containing schizonts as observed in the diclazuril-treated group, showing the anticoccidial effect of SMC by affecting the early schizont stage, similar to that of diclazuril. Damage of the cecal tissue results in release of plasma proteins into the intestine with hemorrhage and absorption disturbance, reducing the TP level in case of coccidiosis. This is in accordance with our results, which could be ameliorated by SMC by interfering with the *Eimeria* life cycle.

In the infected groups treated with SMC (G4 and G5), histopathological examination of cecal tissues showed that the cecal crypts and migrating epithelial cells have necrotic elements of the *E. tenella* schizonts; necrosis occurred in a dose-dependent manner. Furthermore, the alive meronts of *E. tenella* decreased in G4 and G5. The effect of SMC was similar to that of diclazuril (G6), which revealed high dead parasitic stages in the crypts' epithelial cells. The *E. tenella*-induced severe necrotic and hemorrhagic enteritis is in accordance with a previous report showing the existence of different parasitic stages in the intestinal crypts (50). Histopathological results demonstrated that SMC might affect the early schizogony stage, producing an anticoccidial effect. Regarding the induced hepatic damage by *E. tenella* infection, mitochondrial damage was indicated by the elevations of the aminotransferase enzymes (ALT and AST), while the cellular and biliary damage were referred by the elevated ALP activity. Moreover, both TP and albumin were significantly reduced in the infected chickens because of hepatic cell damage, which is responsible for the protein synthesis (51). Significant elevations in creatinine and uric acid levels were also observed because of coccidiosis due to reduced feed and water intake. SMC treatment could restore the ALT, AST, ALP, TP, albumin, creatinine, and uric acid levels toward the normal levels as measured on the 7th and 14th day after infection, revealing the protective effect of SMC, particularly the high dose, which showed a higher protection than diclazuril. The SMC-induced protection is attributed to the organosulfur nature and its antioxidative ability, which reduces hepatic and renal damage by restoring the normal cellular membrane structure and lipid peroxidation inhibition (52, 53).

It is well-known that coccidiosis leads to the release of reactive oxygen species (ROS) because of the damaged tissues (54). As a result, antioxidant enzymes (SOD, GSHPx, and CAT) were highly consumed to overcome the oxidative stress in the infected chickens (G3). SMC treatment (G4 and G5) could restore the activities of the measured antioxidant enzymes toward the normal levels by reducing the cecal and hepatic tissue damage and lipid peroxidation similar to that in diclazuril. These data were in accordance with previous studies that reported the scavenging of free radicals by the organosulfur content of garlic extract (55). Moreover, SMC was able to inhibit the NADPH oxidase enzyme and its signal transduction, leading to reduced ROS release as reported previously (56). The current data of the antioxidant parameters revealed the antioxidant activity of SMC against *Eimeria*-induced oxidative damage. It is noteworthy to state that *E. tenella* infection stimulates the immune cells to express pro-inflammatory cytokines (57). The inflammatory response was indicated by upregulation of NF-κB1 expression in the cecal tissue as observed in G3. NF-κB1 promotes the expression of other cytokines, such as IL-1β and IFN-γ, which activate the signal transduction of NF-κB pathway ending by tissue inflammation as confirmed by histopathological examination. The release of IL-1β was reported previously on the 7th day after coccidia infection (57). In this study, the dysregulated gene expressions of NF-κB1, IL-1β, and IFN-γ were restored to the normal regulation after treatment of infected chickens with SMC by the same significance as that with diclazuril. The anti-inflammatory effect of SMC is probably due to its ability to restrict the phosphorylation of cytochrome p65, reduce the inflammatory cytokine release, and inhibit cyclooxygenase-2 signal transduction, which have a potential role in the inflammatory response (56, 58). These data revealed the anti-inflammatory activity of SMC against the *E. tenella*-induced inflammatory response. Altogether, the recorded oocyst count and biochemical, antioxidant, and anti-inflammatory parameters after treatment of the infected chickens by SMC explain how the clinical signs, lesions, and mortality rate could be improved along with reduced BWG and FCR, which were all induced by *E. tenella* infection, demonstrating the dose-dependent protective role of SMC against coccidiosis effects.

## Conclusion

Taken together, the present study concluded the anticoccidial role of SMC as a plant-based compound against *E. tenella*-induced coccidiosis in broiler chickens. Finally, our conclusion recommends the use of SMC, a garlic component, as a supplementary or alternative therapy to control avian coccidiosis induced by *E. tenella*. Further clinical trials should be conducted to confirm its efficiency, and more studies are needed to determine the detailed mechanism of action of SMCs.

## Data Availability Statement

The original contributions presented in the study are included in the article/supplementary material, further inquiries can be directed to the corresponding author/s.

## Ethics Statement

The animal study was reviewed and approved by the Research, Publication, and Ethics Committee of the Faculty of Veterinary Medicine, Kafrelsheikh University, Egypt. The ethical approval number is KFS-2019/3.

## Author Contributions

EE and WA were involved in the conception of the idea and methodology design, performed data analysis and interpretation, and prepared the manuscript for publication and revision. AF performed data interpretation, wrote the initial draft of the manuscript, and provided scientific revision of the final manuscript. DE, AR, AA, RM, NN, NA, EH, and KA provided scientific advice and prepared the manuscript for revision. All authors have read and approved the final manuscript.

## Funding

The work was supported by Taif University Researchers Supporting Program (project number: TURSP-2020/153), Taif University, Saudi Arabia.

## Conflict of Interest

The authors declare that the research was conducted in the absence of any commercial or financial relationships that could be construed as a potential conflict of interest.

## Publisher's Note

All claims expressed in this article are solely those of the authors and do not necessarily represent those of their affiliated organizations, or those of the publisher, the editors and the reviewers. Any product that may be evaluated in this article, or claim that may be made by its manufacturer, is not guaranteed or endorsed by the publisher.
